# Cold seep seafloor observatories revise the notion of ‘life in the slow lane’

**DOI:** 10.1093/nsr/nwag267

**Published:** 2026-05-14

**Authors:** Andreas Teske

**Affiliations:** Department of Earth, Marine and Environmental Sciences, University of North Carolina at Chapel Hill, USA

**Figure 1.**
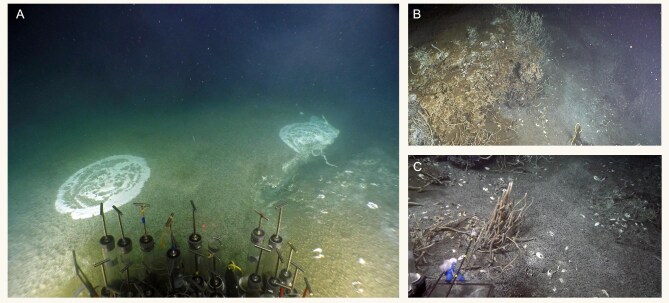
(A–C) Cold seep community with methane-derived carbonates, methane-oxidizing tube worms, chemosynthetic clams, and methane-soaked sediments dominated by methane-oxidizing microbial communities. This example comes from the Octopus Mound cold seeps in Guaymas Basin, Gulf of California (photographs taken by A. Teske).

Research on the dynamics of chemosynthetic deep-sea ecosystems has followed two major directions, the studies of hydrothermal vents and cold seeps. Choosing one or the other has impacted the biological perspective of practitioners in the field: hydrothermal vent researchers are accustomed to rapid and episodic change; cold seep researchers are on the whole attuned to stability and continuity. Comparing basic characteristics of these habitats helps to understand why. Concerning hydrothermal vents, spots of active hydrothermal venting change rapidly along with the meandering underground flow path of hydrothermal fluids, both are constantly reshaped by earthquakes and volcanic eruptions. In response, colonization of hydrothermal habitats by microbes and macrofauna is fast, propagation needs to be effective within a year or two, and life cycles follow a boom-and-bust pattern as old vents are extinguished and new ones are born every few years [1,2]. On the other hand, cold seeps sustain some of the longest-lived invertebrates on the planet; methane- and sulfur-oxidizing Lamellibrachia tubeworms form colonies that are more than a hundred years old [3]. The extended time scales on which cold seep tube worm communities develop and mature have been characterized as ‘life in the slow lane’ [4] (Fig. 1).

This familiar dichotomy between ‘slow’ cold seeps and ‘fast’ hydrothermal vents can no longer be uncritically accepted. In a pioneering multiyear study of a nascent cold seep in the South China Sea, a multi-institutional research consortium led by Prof. Fengping Wang of Shanghai Jiao Tong University has convincingly shown that microbial colonization of seep sediments proceeds surprisingly fast after being triggered by methane hydrate leakage [5]. In a natural seep community, diverse lineages of anaerobic methane-oxidizing archaea (ANME), with their individual adaptations to divergent methane and sulfate supply, are reacting in concert and assemble within 1–2 years into a functional methane-oxidizing community—the microbial methane filter that keeps methane emissions from the seabed under control. Subsequently, the microbial ecosystem plus seep-associated macrofauna matures within the observation period of 6 years. Once established, this new seep is likely to be long-lived, its life span delimited primarily by the underlying methane supply. This multifaceted and thorough study offers hope that the rapid microbial response to newly initiated seepage can keep pace with methane hydrate dissociation in the gradually warming ocean, without lengthy incubation windows of multiple decades that have been predicted based on modelling studies of anaerobic methane oxidation in marine sediments (summarized in ref. [5]), and slow growth of ANME archaea in laboratory enrichments [6]. As a caveat, the reader should remember that marine methane seepage is only a minor proportion of global methane sources, and that anaerobic methane oxidation in terrestrial and freshwater habitats—using electron acceptors other than sulfate—needs to be accounted for as well when predicting the possible extent of microbial control on global methane emissions [7].

Implicitly, Liang and colleagues are calling for renewed and widespread efforts of long-term seabed monitoring, with a focus on the evolution of cold seepage under climate change conditions. The concept of cold seep observatories has existed for ∼20 years. Examples include monitoring of the massive Haakon–Mosby mud volcano and its eruptive activity in the Arctic Ocean [8,9]; the Gulf of Mexico Research Hydrate Consortium and its long-term monitoring of Woolsey Mound, a carbonate/hydrate cold seep in the Gulf of Mexico [10]; and the Gulf of Mexico Research Consortium that relied on this baseline to assess the ecosystem imprint of the Deepwater Horizon oil spill in the very same habitat [11]. Beyond the particular scientific objectives of cold seep research, long-term monitoring of these ecosystems will be an essential tool of assessing ocean health in the future. Liang and colleagues have demonstrated a comprehensive strategy for cold seep monitoring that should be implemented more widely.
